# Temporary Dorsal Staple Fixation of Scapholunate Interosseous Ligament Repair and Reconstruction

**DOI:** 10.1016/j.jhsg.2025.100891

**Published:** 2025-12-05

**Authors:** Rafa Rahman, Matthew V. Abola, Michelle G. Carlson

**Affiliations:** ∗ Division of Hand and Upper Extremity, Hospital for Special Surgery, New York, NY

**Keywords:** Carpal instability, Scapholunate interosseous ligament, Staple

## Abstract

There is a wide variety of techniques to address scapholunate interosseous ligament injury, including both repair and reconstruction of the ligament. What many of these techniques have in common is the protection of the repair or reconstruction by the use of Kirschner wires (K-wires) temporarily placed across the scapholunate and sometimes scaphocapitate articulations to provide immobilization. There are multiple potential downsides to K-wire utilization, including possible interference with the repair or reconstruction, distraction of the scapholunate articulation as the K-wire is passed, occasional need for multiple passes for proper placement, contribution to stress risers within the bone, and unintentional K-wire complications, including breakage, migration, and infection. We describe the use of a dorsal, partially-inserted nitinol staple at the scapholunate articulation as an improved technique over K-wire use for temporary immobilization of the joint. Utilization of the staple allows for compression of the scapholunate interval, direct visualization during insertion, and the ability to avoid interference with the scapholunate interosseous ligament repair or reconstruction. In addition to a description of our surgical technique, we provide a summary of our experience using this technique in patients and a case illustration.

The scapholunate interosseous ligament (SLIL) is a critical stabilizer of the wrist, and injury to the SLIL can result in wrist instability and arthritis if left untreated.^1^^,^^2^ While SLIL injury is not an uncommon cause of carpal instability, there exists a multitude of surgical treatment options, including both repair and reconstruction, and approaches include both arthroscopic and open techniques.^2^ Regardless of the chosen surgical treatment, most require protection of the repair or reconstruction through temporary immobilization of the scapholunate articulation, and in some instances also the scaphocapitate articulation.^2^ One survey of over 400 hand surgeons found that 99% use Kirschner wire (K-wire) immobilization in acute scapholunate instability and 94% in chronic scapholunate instability. The majority also use both scapholunate and scaphocapitate K-wires in the acute and chronic settings.^3^

Despite the prevalence of K-wire utilization for temporary immobilization after SLIL repair or reconstruction, there are numerous potential downsides. SLIL repair or reconstruction may use anchors or bone tunnels placed in the scaphoid and/or lunate.^2^ The surgeon must exercise extreme caution to avoid interference of the K-wire with the anchors or tunnels, as this may compromise the repair or reconstruction construct. While K-wire placement in the volar half of the carpus should not interfere with dorsally placed anchors, this would be insufficient for techniques that use dorsal and volar fixation, or rely on bone tunnels.^4^ Difficulty in obtaining the correct trajectory through a narrow corridor of bone may lead to multiple attempts at passing the K-wire. This, as well as remaining pin tracks once even adequately placed K-wires are ultimately removed, may create stress risers and potential for postoperative fracture, as has been noted in other hand surgery applications.^5^ As the K-wire is passed across the scapholunate articulation, one may see distraction between the scaphoid and lunate. While reduction of the articulation while passing the K-wire may mitigate this issue, once placed, the K-wire does not provide any compressive force across the scapholunate interval. Finally, K-wires are commonly associated with complications in hand and wrist surgery.^5^ There is the possibility of K-wire migration, loosening, irritation of surrounding structures, infection, bending, or breakage.^5^, ^6^, ^7^ Migration, loosening, and breakage of the K-wire not only loses the advantage of proper immobilization but can also damage surrounding structures. The risk of bending, breakage, and migration of K-wires can be decreased by proper immobilization prior to K-wire removal. Specific to scapholunate stabilization, K-wires inserted radially to ulnarly can irritate overlying tendons, superficial sensory branches of the radial nerve, or the radial artery. This particular complication can be mitigated by making a small radial incision and bluntly dissecting to bone, however, thus necessitating an additional incision. Following SLIL repair and reconstruction, one study found that among the most common causes of complication were issues related to the use of K-wires and that those with postoperative complications warranting additional surgery ultimately had worse outcomes including greater pain and weaker grip strength, and worse radiographic parameters.^7^

In light of the limitations of K-wire immobilization following surgery for SLIL injury, we describe a technique using a temporarily placed, partially-inserted nitinol staple for protecting the SLIL repair or reconstruction. The staple is inserted dorsally at the scapholunate articulation under direct visualization, thus affording easy placement and greater ability to avoid interference with the SLIL repair or reconstruction anchors or tunnels. Avoidance of the repair/reconstruction is also aided by the fact that the staple is intentionally left proud, to aid in later removal. Additionally, for this reason, a dorsal capsulodesis or repair can be performed deep to the staple and not disturbed with later staple removal. When the primary SLIL repair or reconstruction is performed from a dorsal approach to the wrist, the use of the staple does not result in any additional incisions. The staple also allows for compression of the scapholunate interval, given its biomaterial properties. Nitinol staples are an alloy of nickel and titanium, thus retaining temperature-dependent elastic properties, commonly referred to as a “shape memory” alloy.^8^ The nickel and titanium alloy is 16 to 32 times more elastic than other alloy combinations and provides the ideal properties for achieving compression at the site across which the staple is placed. “Shape memory” alloy staples have inherent compressive properties such that after insertion of the staple across the desired area of compression, the metal will return to its original orientation.^9^ For some staple designs, this may be facilitated in part by the body’s warmer temperature compared to the surrounding ambient temperature, which causes the staple tines to compress and then maintain uniform compression.^8^^,^^9^ It should be noted that the exact amount of compression provided by the staple cannot be controlled by the surgeon, and is thus a potential limitation of this technique. However, the compressive property of the staple is advantageous over K-wire use, which does not afford any compression. Several biomechanical studies have shown staples to be superior to retained K-wires in controlling carpal motion. In one study of 42 pairs of cadaveric scapholunate articulations, fixation was randomized to two K-wires, a 3-mm screw (simulating the reduction and association of scapholunate technique), and either two- or four-tine staples. In all cases, the four-tine staples demonstrated superior rotational control.^10^ In another study, range of motion was examined in eight cadaveric wrists with intact SLIL, sectioned SLIL, or scapholunate interval immobilized with K-wire or staple fixation. Staple fixation demonstrated preservation of nearly physiologic range of motion without compromise of stability.^9^

In this surgical technique guide, we describe our method for using a dorsally placed, partially-inserted nitinol staple to temporarily immobilize the scapholunate articulation following SLIL repair or reconstruction. We also share our experience using this technique in patients and provide a case illustration.

## Indications and Contraindications

This technique is indicated in patients who are undergoing SLIL repair or reconstruction and require temporary postoperative immobilization of the scapholunate articulation. The technique is best used in cases where the SLIL repair or reconstruction is being performed through an open dorsal approach.^10^ However, the staple can still be inserted through a separate mini-open dorsal approach to the carpus if a different surgical approach is used for the SLIL repair or reconstruction. Use of the staple is contraindicated if the site of staple placement would interfere with the SLIL repair or reconstruction fixation, or in a patient with limited bony real estate for staple placement. The staple should also not be used if a patient has an allergy to a component of the staple alloy. Interestingly, Johnston et al^11^ demonstrated a positive correlation between bone mineral density (BMD) and SLIL ultimate load. Staple fixation may theoretically be influenced by BMD, with osteopenic or osteoporotic patients at greater risk for loosening, and thus BMD should be considered in patient selection.

## Surgical Anatomy

The SLIL is a crucial stabilizer of the wrist and consists of three distinct components: dorsal, proximal, and volar. The dorsal component is the thickest and provides at least as much, if not greater, mechanical strength compared to the proximal component, making it a primary focus of repair and reconstructive techniques.^1^^,^^12^ This anatomy is replicated in international populations, with a study of Vietnamese wrists demonstrating that even though overall SLIL size was smaller compared to studies on Western populations, the dorsal portion was still thickest relative to the other components of the SLIL.^13^ Thus, our staple insertion technique is described from a dorsal approach. The same Vietnamese study also reported a lower average SLIL tensile strength compared to prior studies on Western populations. Nonetheless, our described staple utilization should provide adequate temporary immobilization irrespective of differences in SLIL strength, making it an adaptable technique.^13^ Either a ligamentous splitting or reverse T-arthrotomy may be used to access the dorsal carpus, and one must take care to preserve or repair the dorsal ligament complex, which includes the dorsal intercarpal and dorsal radiocarpal ligaments.^1^ One should also note the size of the scaphoid and lunate, as this impacts the staple size. The legs of the staple should be chosen such that they traverse at least 50% of the bone in the volar-dorsal dimension, while still allowing the bridge of the staple to sit proud.

## Surgical Technique

1. Before surgery, in addition to the standard discussion of risks, benefits, and alternatives, the patient should be made aware of the planned return to the operating room for hardware removal, which can be performed with local anesthesia and sedation.
2. This technique can be performed with either a transverse or longitudinal skin incision. The senior author’s preference is a transverse incision of approximately 3 cm length, centered over the scapholunate interval and within a Langer’s line (Fig. 1A). A dorsal approach is then performed through the third and fourth extensor compartments, using Lister’s tubercle as a landmark.

**Figure 1:**
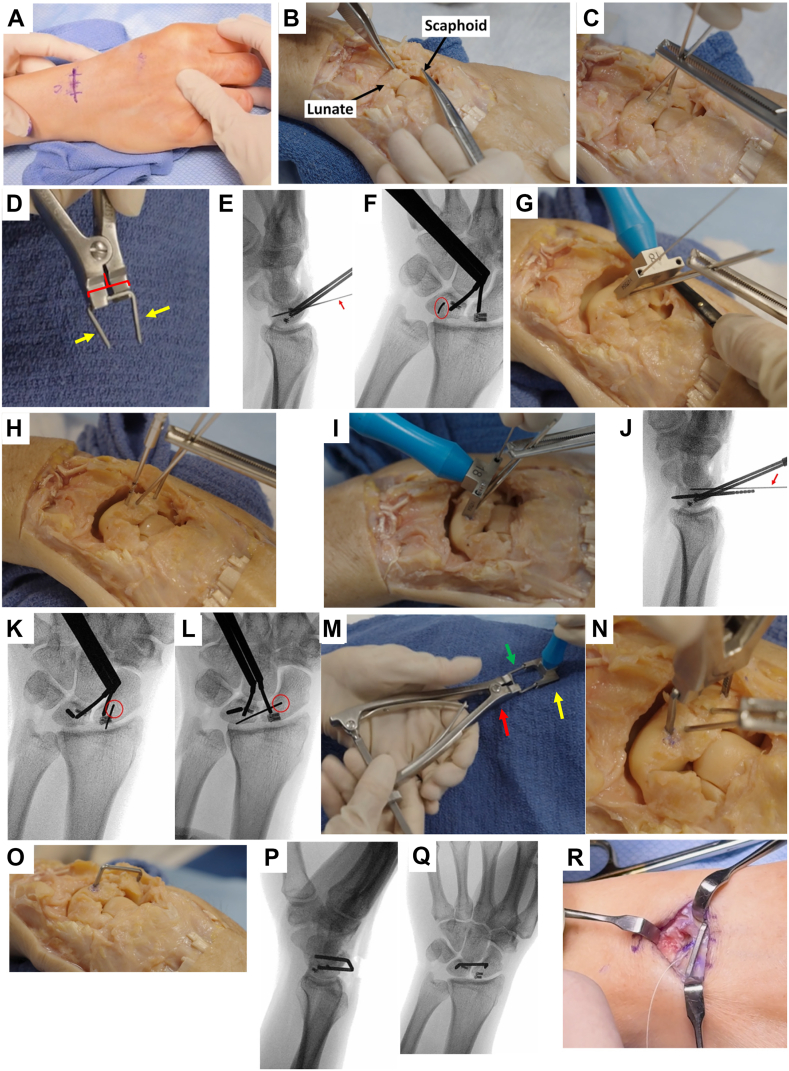
Technique for dorsally-inserted nitinol staple for temporary scapholunate immobilization. Technique is demonstrated both in live patient with intraoperative clinical and fluoroscopic images (**A, E, F, J−L, P−R**), as well as on cadaveric specimen with extensile exposure of the carpus for demonstration purposes (**B−D, G−I, M−O**). **A** 3-cm skin incision centered over scapholunate interval and within a Langer’s line. Following, this, a dorsal approach is performed through the third and fourth extensor compartments. A dorsal capsulotomy is made to expose the scapholunate interval and SLIL injury. **B** Dorsal scaphoid translation can often be appreciated at this point. **C** One 1.6 mm Kirschner wire (K-wire) is placed in the scaphoid and one in the lunate as joysticks, and following repair or reconstruction of the SLIL, these joystick wires can be used to maintain the reduction. This can be performed with a Kocher clamp (shown here) or with a cannulated reduction clamp. **D** A staple of the appropriate size bridge (red bracket) and legs (yellow arrows) is chosen. **E** A K-wire is placed at the intended position of the lunate leg of the staple and position confirmed fluoroscopically on lateral and **F** posteroanterior imaging. **G** The staple guide is used to confirm that the scaphoid leg of the staple will sit in the appropriate position. **H** The lunate K-wire is over-drilled using a cannulated drill. Following drilling, a peg is placed in the drilled hole in the lunate and the staple guide held in place, while **I** a K-wire is placed in the intended position of the scaphoid leg of the staple. Position is confirmed fluoroscopically on **J** lateral, **K** oblique, and **L** posteroanterior imaging. **M** The nitinol staple (green arrow) is placed in the introducer (red arrow), and confirmed that it fits in the guide (yellow arrow). **N** The staple is inserted into the drill holes and malleted into place, **O** intentionally left proud for ease of later removal. The joystick K-wires are removed. Staple placement is confirmed on **P** lateral and **Q** posteroanterior fluoroscopic imaging. **R** The capsule is closed deep to the staple, and may require a t-ing transversely.
3. A dorsal capsulotomy is made to expose the scapholunate interval and SLIL injury. Often, dorsal scaphoid translation can be appreciated (Fig. 1B).
4. One 1.6 mm K-wire is placed in the dorsal scaphoid in a dorsal to volar trajectory, and one 1.6 mm K-wire is placed in the dorsal lunate in a dorsal to volar trajectory, to be used as joysticks to aid in mobilization and reduction.^14^ These will be later removed prior to completion of the case and are not necessarily essential for staple placement. The surgeon also has great latitude in where these K-wires can be placed to avoid any potential interference with the repair or reconstruction fixation.
5. The surgeon should then perform their preferred SLIL repair or reconstruction technique. There is no difference in the staple insertion technique when performing a primary repair versus reconstruction. When performing a reconstruction, the senior author’s preferred method uses all-suture, knotless anchors placed in the scaphoid and lunate, with repair stitches shuttled into the opposing anchor and then tensioned.
6. Following repair or reconstruction, the scapholunate interval can be held reduced with a Kocher clamp or cannulated reduction clamp around the joy stick K-wires (Fig. 1C).
7. A staple of the appropriate size bridge and legs are selected. The bridge should be selected such that the two legs sit equidistant from the scapholunate interval, and that leg placement will not interfere with repair or reconstruction fixation. The legs should be long enough to traverse at least 50% of the volar-dorsal length of the scaphoid and lunate in order to perform compression along the scapholunate interval, and to also allow the staple to sit at least 5 mm proud after insertion (Fig. 1D).
8. A K-wire is placed at the intended position of the lunate leg of the staple, orthogonal to the SLIL. Positioning should be confirmed fluoroscopically (Fig. 1E, F). Note that this K-wire is simply used to guide the position of the staple leg and will not be retained. The staple guide should also be used to confirm that the scaphoid leg of the staple will ultimately sit in the appropriate position (Fig. 1G).
9. The lunate K-wire is then over-drilled using a cannulated drill (Fig. 1H). The drill can be marked at 10 and 15 mm to give the surgeon a sense of the depth being drilled. There is no need to penetrate the volar cortex. A peg is placed in the drilled hole, with the staple guide in place.
10. A K-wire is placed through the guide in the intended position of the scaphoid leg of the staple, orthogonal to the SLIL (Fig. 1I). Positioning is confirmed fluoroscopically (Fig. 1J−L). Note that this K-wire is also simply used to guide the position of the staple leg and will not be retained.
11. The nitinol staple is placed in the introducer, and the surgeon should confirm that it fits the guide (Fig. 1M). The staple is then inserted into the drilled holes and malleted into place (Fig. 1N), and intentionally left 5 mm proud from the dorsal cortex of the scaphoid and lunate for later ease of removal (Fig. 1O). Placement is confirmed fluoroscopically (Fig. 1P, Q). The joystick K-wires can now be removed. At this point, no K-wires remain in the carpus.
12. The capsule is closed deep to the staple, and may require a T-ing transversely (Fig. 1R).

## Postoperative Management

Following staple placement, the patient is immobilized in a thumb spica cast or splint for 10 weeks, at which point the staple is removed and a gentle range of motion is initiated. For staple removal, the prior incision is used, after local anesthesia and sedation. Dissection is performed directly over the staple, which is removed by cutting the bridge, and then each limb of the staple is removed independently. Dissection need not be performed deep to the repaired capsule, as the staple would have been intentionally left proud to the capsule to allow for ease of removal. Thus, removal should not violate the SLIL repair or reconstruction. One must acknowledge that removal of a radially placed K-wire would not require additional surgery in the area of repair/reconstruction at all, although safe placement would have necessitated a separate incision at the time of placement during the index surgery.

## Pearls and Pitfalls

1. A bump placed volarly under the wrist during surgery will aid in the dorsal exposure of the scapholunate interval.
2. When placing the lunate joystick K-wire, use caution to not penetrate the concave midcarpal joint (Fig. 2A). We recommend placing the wire at the edge of the radiocarpal articulation to avoid this issue (Fig. 2B).

**Figure 2:**
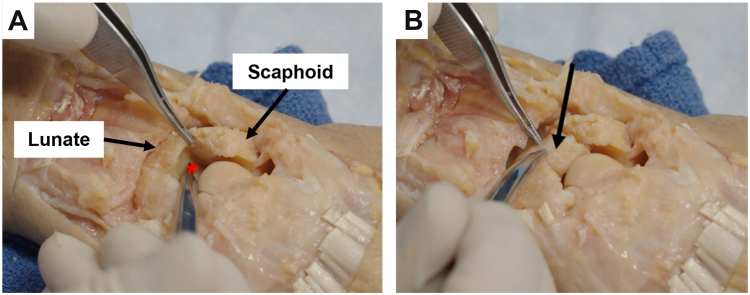
Avoid placement of lunate joystick Kirschner wire (K-wire) in midcarpal joint. **A** Red asterisk indicates concave lunate articulation at the midcarpal joint. **B** By placing the K-wire at the edge of the radiocarpal articulation on the lunate, denoted by the black arrow, one can avoid penetration of the midcarpal joint.
3. We prefer placing the K-wire for the lunate before the K-wire for the scaphoid, as lunate K-wire placement tends to be relatively more technically challenging compared to scaphoid K-wire placement when using the mini-open approach.
4. Many staple systems provide options where one staple leg is longer than the other. We find that this longer leg is usually best placed in the lunate. For both legs, we find that a length of 15 mm at a minimum is usually necessary.

## Complications

Nitinol staples can theoretically loosen, similar to K-wires, and irritate overlying structures, such as the adjacent extensor tendons. We noted staple loosening in one case in our experience. However, the dorsal surface of the staple is smooth, unlike a buried K-wire, and we have not witnessed irritation of overlying tendons with this short-term placement. It would be elucidating for further research to study the rate of loosening compared between K-wires and staples. The usual infection-related risks of hardware usage are also possible with staples. Theoretically, especially in dorsally based repairs or reconstructions, the additional placement of the staple and K-wire joysticks could weaken or fracture the scaphoid or lunate. While this has not been observed in our patient cohort, one may be able to reduce this risk by ensuring adequate spacing between each item, made easier by performing the technique under direct visualization. We also do not recommend using this technique in an excessively small wrist with limited dorsal surface area on the scaphoid and lunate, or in a wrist with poor bone quality. The bridge of the staple should be selected so it is long enough to avoid the repair/reconstruction hardware or tunnels. It is also to the surgeon’s discretion whether to use the joystick K-wires, as this is not essential for the use of the staple, and is simply a tool to aid in the reduction of the scapholunate interval. Overall, staple use has been associated with a low complication profile and has been well-tolerated in our patients.

## Case Illustration

In our experience with 21 wrists in 20 patients with mean follow-up of 31.1 weeks (range, 11.6−65.3 weeks), we have found promising results. All of these patients had sustained SLIL injury and one was in the context of perilunate dislocation. In 14 cases, a supplemental K-wire was placed in the scaphocapitate articulation for additional stabilization, a practice we have not employed in more recent cases as we have found the staple to provide adequate immobilization. One patient had two staples placed, one in the scapholunate and one in the scaphocapitate interval. All patients did well in the immediate postoperative period, with maintained stability of the carpus during the period of immobilization. Staples were maintained for a mean of 10 weeks (range, 9−13 weeks), and patients maintained carpal alignment after staples were removed and were able to initiate range of motion and strengthening. One patient had their staple back-out, and it was replaced. Another patient returned to the operating room for irrigation and debridement because of concern for infection, which was found to be negative.

We also explored outcomes in patients with at least 4 months of follow-up. This timepoint was selected as this is when strengthening was generally initiated. We excluded patients who did not meet this follow-up, and those who had symptoms because of unrelated hand pathologies. Fifteen of 21 patients had at least 4 months of follow-up. Three of these patients were excluded because of unrelated hand pathologies: one had Dupuytren disease, one had pain related to scaphotrapeziotrapezoid degeneration, and one had nonspecific soreness in both radial and ulnar aspects of the hand radiating to the forearm as well as all fingers. Thus, 12 patients were included. A positive outcome was defined as having at least 60° of wrist flexion and 60° of wrist extension, as well as no pain. Ten of the 12 patients had a positive outcome. Of these, six had a supplemental K-wire used in addition to the staple, and four did not. The two patients who did not have a positive outcome per our definition both had a supplemental K-wire used. One of these patients lacked 20° of flexion, but had returned to playing tennis, had no complaints, and had no further interventions required. The other required continued efforts to improve range of motion. Thus, based on this analysis, we did not find any difference in experience when employing supplemental K-wire fixation versus staple alone for temporary immobilization. While a limitation of our experience is the lack of long-term radiographic data to assess scapholunate interval, all three patients who had at least 1 year of follow-up from the index surgery had promising clinical results. Two patients had full range of motion and no pain, and the third is the patient described above, who lacked 20° of flexion but had otherwise returned to activity with no complaints. Institutional review board approval was obtained to share this data.

**Figure 3:**
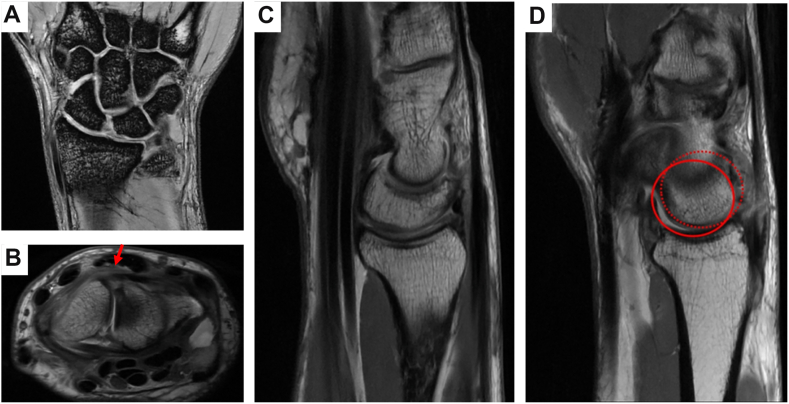
Magnetic resonance imaging of wrist with scapholunate interosseous ligament (SLIL) injury. **A** Coronal, **B** axial, and **C, D** sagittal imaging demonstrating disruption of the SLIL. **B** Disruption of the SLIL denoted by the red arrow. **C** Dorsal tilt of the lunate suggests dorsal intercalated segment instability. **D** Dorsal scaphoid translation.

We illustrate our technique in a man with right wrist pain and swelling after a fall while playing tennis. He presented 1.5 months following the injury with pain in wrist extension and was unable to perform a push-up. On physical examination, he was tender to palpation at the dorsal wrist, lacked 10° of wrist extension, and had a positive scaphoid shift test. MRI demonstrated disruption of the SLIL (Fig. 3A, B), dorsal tilt of the lunate (Fig. 3C), and dorsal scaphoid translation (Fig. 3C), together suggesting dorsal intercalated segment instability. He was indicated for SLIL repair. Through a dorsal approach and inverse-T capsulotomy, the scapholunate interval was exposed. Repair of the SLIL was performed using a suture anchor technique, with two anchors in the scaphoid and one in the lunate. Following this repair, an 18 mm (bridge) × 14 mm (leg) × 14 mm (leg) staple was placed to immobilize the scapholunate interval using the above-described technique (Fig. 1P, Q). The patient was immobilized for a total duration of 10 weeks, at which point the staple was removed and a gentle range of motion initiated. The patient did well after surgery. Written informed consent was obtained from the patient for publication of this case illustration and accompanying images. The study was also approved by our institutional review board.

## Conflicts of Interest

Dr Carlson is a consultant for Stryker. In-kind donation of staples was provided by Stryker; however, Dr Carlson has no association or financial involvement with this product. We do not believe this declaration of interest to have any influence on our work, and do not mention any specific companies in our manuscript. No benefits in any form have been received or will be received by the other authors related directly to this article.

## References

[1] Wessel L.E., Wolfe S.W. (2023). Scapholunate instability: diagnosis and management - anatomy, kinematics, and clinical assessment - Part I. J Hand Surg Am.

[2] Zhou J.Y., Jodah R., Joseph L.P., Yao J. (2024). Scapholunate ligament injuries. J Hand Surg Glob Online.

[3] Zarkadas P.C., Gropper P.T., White N.J., Perey B.H. (2004). A survey of the surgical management of acute and chronic scapholunate instability. J Hand Surg Am.

[4] Sandow M., Fisher T. (2020). Anatomical anterior and posterior reconstruction for scapholunate dissociation: preliminary outcome in ten patients. J Hand Surg Eur Vol.

[5] Hsu L.P., Schwartz E.G., Kalainov D.M., Chen F., Makowiec R.L. (2011). Complications of K-wire fixation in procedures involving the hand and wrist. J Hand Surg Am.

[6] Schmidt G.J., Dent C., Nguyen A., Nydick J. (2025). Early Postoperative outcomes of surgical fixation of proximal phalanx fractures with intramedullary nails versus Kirschner wires. Hand (N Y).

[7] Rohman E.M., Agel J., Putnam M.D., Adams J.E. (2014). Scapholunate interosseous ligament injuries: a retrospective review of treatment and outcomes in 82 wrists. J Hand Surg Am.

[8] Farr D., Karim A., Lutz M., Calder J. (2010). A biomechanical comparison of shape memory compression staples and mechanical compression staples: compression or distraction?. Knee Surg Sports Traumatol Arthrosc.

[9] Hess D., Archual A., Burnett Z. (2021). Motion and strength analysis of 2-tine staple and K-wire fixation in scapholunate ligament stabilization in a cadaver model. J Hand Surg Glob Online.

[10] Toby E.B., McGoldrick E., Chalmers B., McIff T. (2014). Rotational stability for intercarpal fixation is enhanced by a 4-tine staple. J Hand Surg Am.

[11] Johnston J.D., Small C.F., Bouxsein M.L., Pichora D.R. (2004). Mechanical properties of the scapholunate ligament correlate with bone mineral density measurements of the hand. J Orthop Res.

[12] Nikolopoulos F.V., Apergis E.P., Poulilios A.D., Papagelopoulos P.J., Zoubos A.V., Kefalas V.A. (2011). Biomechanical properties of the scapholunate ligament and the importance of its portions in the capitate intrusion injury. Clin Biomech (Bristol).

[13] Le T.N., Do H.P., Nguyen P.D. (2025). Anatomy and biomechanics of the scapholunate ligament in Vietnamese cadavers. JPRAS Open.

[14] Kang L, Dy CJ, Wei MT, Hearns KA, Carlson MG (2018). Cadaveric testing of a novel scapholunate ligament reconstruction. J Wrist Surg.

